# Revisiting Systemic Thrombolysis in Acute Pulmonary Embolism

**DOI:** 10.1016/j.jacadv.2024.100923

**Published:** 2024-03-26

**Authors:** Zach Rozenbaum

**Affiliations:** Department of Cardiology, Tulane University, New Orleans, Louisiana, USA

**Keywords:** aspiration devices, pulmonary embolism, systemic thrombolysis

Systemic thrombolysis (ST) for acute pulmonary embolism (PE) has been studied in numerous trials, and evidence for its use remains debatable. Data are not lacking but rather failed to demonstrate an overall convincing risk-benefit ratio. The most robust study, representing today’s practice, is the Fibrinolysis for Patients with Intermediate-Risk Pulmonary Embolism trial, which showed no mortality benefit with ST for intermediate-high-risk PE.^1^ Moreover, 1 of every 9 patients had major bleeding,^1^ and there was even no long-term pulmonary hypertension reduction.^2^ In unstable patients who have more to gain from ST, the bleeding risk is increased due to several mechanisms, such as venous congestion affecting the liver and gastrointestinal tract and increased right atrial pressure enabling small paradoxical emboli via patent foramen ovale that may undergo hemorrhagic conversion. Meta-analyses showed that ST is not beneficial for stable patients.^3^ While ST reduces the mortality risk in unstable patients (number needed to treat = 59), it increases major bleeding (number needed to harm = 18), including intracranial bleeding (number needed to harm = 78).^4^ Younger patients have a lower risk of major bleeding^4^; however, over half of patients with high-risk PE are not treated with ST because of a perceived increased risk of bleeding.^5^ Consequently, the rate of ST use is only 2.5% overall and 11% in patients with high-risk PE.^6^ Similar to intermediate-risk PE, there are no robust data supporting the use of ST in high-risk PE. Studies examining the mortality benefit of ST in high-risk PE have not been consistent, and the consensus for the practice is based on a trial that consisted of 4 patients in the treatment arm.^7^ Of note, the agent that was given was streptokinase, which is less commonly used nowadays compared to Alteplase. Accordingly, mortality rates in patients with PE remain high, and over the course of 20 years, there has been no clear reduction in the trend of overall PE mortality.^8^

The probable reason ST is still recommended as first-line therapy in PE patients with high-risk features is the lack of randomized controlled trials (RCTs) of newer therapies. Catheter-based therapies, such as percutaneous aspiration devices (PASDs), became available in recent years and are being utilized more commonly in all spectrums of PE risk profiles.^9^ There are over a 1,000 published cases of PASD use, and many more unpublished cases were likely performed. An example of the main part of a saddle PE removed as a single unit using a PASD is shown in Figure 1. Registry data show that the risk of major procedural complications with PASDs is <1%.^10^ Similarly, the 30-day mortality rate with PASD is <1%. Moreover, results of the FLowTriever for Acute Massive Pulmonary Embolism study demonstrated an over 90% in-hospital mortality reduction compared to the 29.5% mortality rate seen in patients treated with other therapies.^11^ Additional areas of potential benefit include long-term complications such as chronic thromboembolic pulmonary hypertension (CTEPH) and post-PE syndrome. CTEPH and post-PE syndrome may occur in up to 6% and almost 50% of patients with PE, respectively.^5^ In the FlowTriever All-Comer Registry for Patient Safety and Hemodynamics study, CTEPH was found in only 1.2% of patients at follow-up.^10^

**Figure 1:**
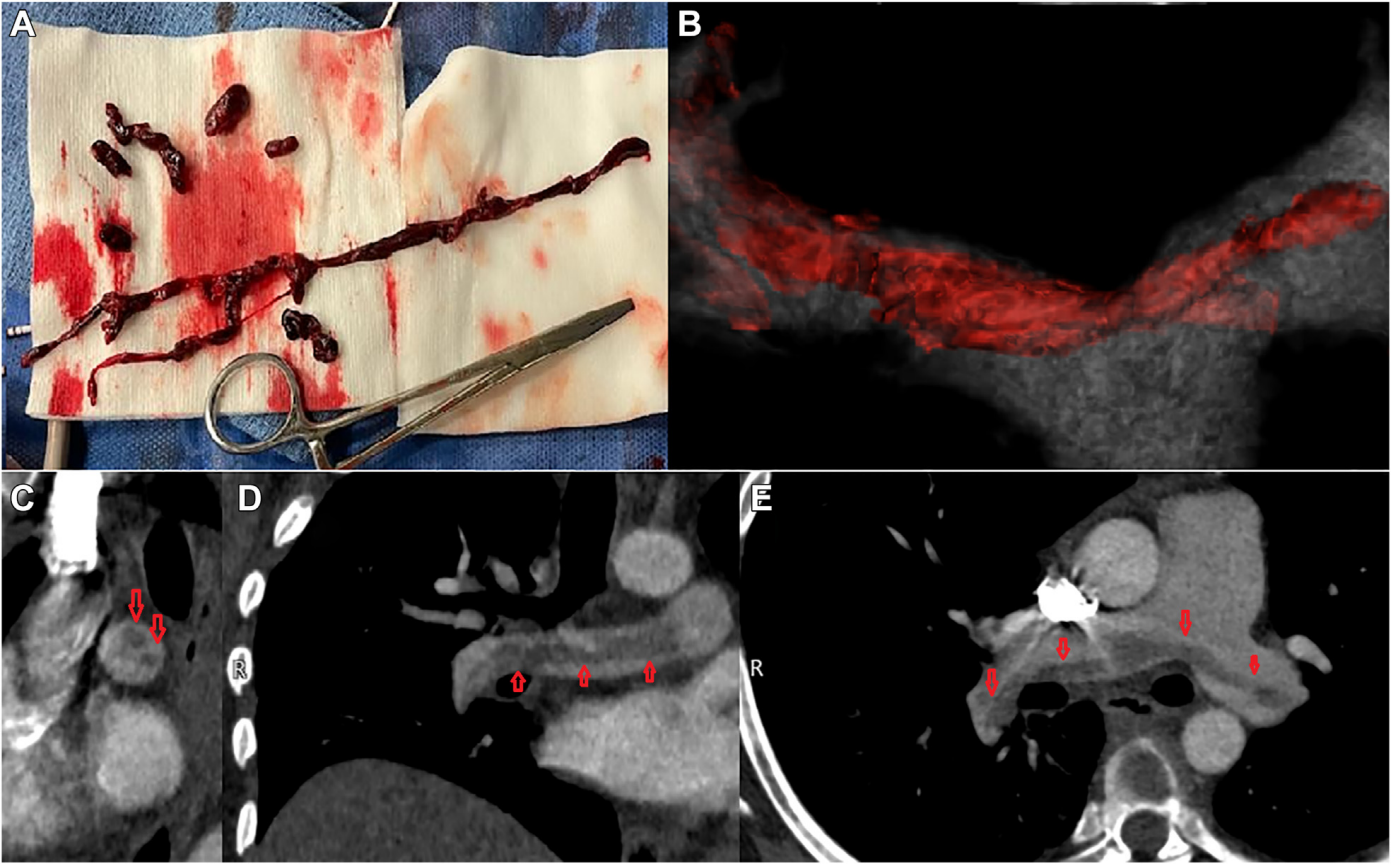
**Saddle Pulmonary Embolism Before and After Aspiration** (A) The aspirated material. The main part of the thrombus was removed as a single unit, and additional small fragments are seen. (B) 3D reconstruction of the saddle pulmonary embolism. (C) Sagittal view of the pulmonary embolism. Red arrows mark the 2 ends. (D) Coronal view of the embolism in the right pulmonary artery, marked by red arrows. (E) Axial view of the pulmonary embolism extending bilaterally, marked by red arrows.

There are many therapies in medicine that are not supported by RCTs. For example, conducting a RCT of diuretic therapy vs placebo in patients with pulmonary edema is deemed unethical but remains standard of practice. Likewise, most surgical operations are not based on RCTs. RCTs of PASDs are anticipated, but it may be years until results are published. In the meantime, PASDs are becoming first-line therapy in many centers for the simple reason that we observe high efficacy and low rates of major complications. ST has been used for PE for over 50 years, despite a lack of convincing evidence. In the era of new effective technologies for PE treatment with low procedural risk, for many, the use of ST is becoming unethical.

## Funding support and author disclosures

Dr Rozenbaum has received a consultant fee from Angiodynamics.
